# Editorial: Virtual Plants: Modeling Plant Architecture in Changing Environments

**DOI:** 10.3389/fpls.2016.01734

**Published:** 2016-11-22

**Authors:** Hartmut Stützel, Katrin Kahlen

**Affiliations:** ^1^Institute of Horticultural Production Systems, Leibniz Universität HannoverHannover, Germany; ^2^Institute of Vegetable Crops, Hochschule Geisenheim UniversityGeisenheim, Germany

**Keywords:** plant architecture, FSMP, crop model, Ecophysiology, simulation models

There is increasing awareness that crop productivity is not only a function of the interaction between plants and their environment, but is also determined by the interplay between form and function. The need to better understand and even quantify this complex interaction has led to a new category of models of plant growth and development, often named virtual plants or functional-structural plant models (FSPM, Room et al., 1996; Prusinkiewicz, 2004; DeJong et al., 2011). In contrast to most traditional plant growth models, virtual plants explicitly describe the three-dimensional structure of plants.

This issue is essentially about new approaches to quantify, explain, predict and eventually manipulate the trilateral interaction between plant function, structure and environment. Renton in his opinion paper relates these factors to the first three of the Aristotelian “causes,” his framework for explaining why things are as they are: function, Aristotle's “causa materialis,” describes the change of matter through transport and transformation, structure, Aristotle's “causa formalis” the change in form, and the effects of the environment, his “causa efficientis.” Renton adds Aristotle's fourth cause, the “causa finalis,” asking the “why” question, fundamental in science, and sees FSPMs as heuristic tools in an evolutionary sense.

Central in this respect are **morphogenetic processes** on the organ level, like leaf expansion or internode elongation as presented in the paper of Demotes-Mainard et al. who analyze the inter-plant variation of a rose variety with respect to these processes. In cereals, the formation of side shoots, i.e., tillers, is an important mechanism to regulate stem and ear density, and senescence of individual tillers determines their productive phase. Evers and Vos review approaches to model tillering based on environmental cues or physiological conditions and show how architectural models can also serve to test hypotheses about the effects of signaling chemicals and substrate transport. The architecture of the vascular system of the vascular system determines water and solute transport. Hölttä et al. show in their model analysis that the Münch hypothesis explains phloem transport across organs and even over long distances in tall trees. Beyer et al. also leave the level of the individual organ and model canopy development based on local leaf density. Their simulations of crown growth dynamics demonstrate the inherent dynamic properties of self-organization and adaptation of the proposed framework of partial differential equations. In addition, Shapiro et al. provided insight on plant morphodynamics at the cellular level. Their computational framework can be used for simulations of plant tissue including cell growth and cell division.

Morphogenetic processes directly affect **light interception** at the organ and canopy level. Hofmann et al. show how a simple Monte Carlos-based model of radiation partitioning in vineyards can be used together with a water balance as a component of a growth model to evaluate the risks of climate change to grape production. Light is also the driving force in interplant competition reviewed in the article of Ford. His review assesses the role of plant architecture in interplant competition for light by focusing on both, the dynamics of stands undergoing competitions and the single plant as competitor. He develops a theory for the effects of plant architecture on competition and highlights the role of functional-structural plant models for simulating interplant competition. De Visser et al. analyze the effects of different plant morphologies and light regimes on light interception and light use efficiency, giving an example of the application of an FSPM approach not only for systems understanding, but also for systems control. Buck-Sorlin and Delaire widen the picture and analyze the prospects of FSPM in horticulture, a section of agriculture with a wide spectrum of crops and production systems where manipulation of growth through changing plant morphology by training and pruning is common practice.

Models are by definition simplified representations of reality with simplicity and parsimony being guiding principles in modeling. Models of plant structure and functions are usually detailed and complex. As Renton puts it: “…the strength of FSPMs, their dynamic realism, is also their weakness, because it makes them relatively complex…”. Indeed, a more systematic understanding of the relationships between increasing model complexity and scientific gain would be desirable.

## Author contributions

The editorial for the topic “Virtual plants: Modeling plant architecture in changing environments” was jointly written by KK and HS.

### Conflict of interest statement

The authors declare that the research was conducted in the absence of any commercial or financial relationships that could be construed as a potential conflict of interest.
